# Extracts of Clove (*Syzygium aromaticum*) Potentiate FMSP-Nanoparticles Induced Cell Death in MCF-7 Cells

**DOI:** 10.1155/2018/8479439

**Published:** 2018-08-23

**Authors:** Firdos Alam Khan, Sultan Akhtar, Dana Almohazey, Munthar Alomari, Sarah Ameen Almofty

**Affiliations:** ^1^Department of Stem Cell Biology, Institute for Research and Medical Consultations, Imam Abdulrahman Bin Faisal University, Post Box No. 1982, Dammam 31441, Saudi Arabia; ^2^Department of Biophysics, Institute for Research and Medical Consultations, Imam Abdulrahman Bin Faisal University, Post Box No. 1982, Dammam 31441, Saudi Arabia

## Abstract

Both nanoparticles and cloves (Syzygium aromaticum) possess anticancer properties, but they do not elicit a significant response on cancer cells when treated alone. In the present study, we have tested fluorescent magnetic submicronic polymer nanoparticles (FMSP-nanoparticles) in combination with crude clove extracts on human breast cancer cells (MCF-7) to examine whether the combination approach enhance the cancer cell death. The MCF-7 cells were treated with different concentrations (1.25 *μ*g/mL, 12.5 *μ*g/mL, 50 *μ*g/mL, 75 *μ*g/mL, and 100 *μ*g/mL) of FMSP-nanoparticles alone and in combination with 50 *μ*g/mL crude clove extracts. The effects of FMSP-nanoparticles alone and combined with clove extracts were observed after 24 hrs and 48 hrs intervals. The response of FMSP-nanoparticles-treated cells was evaluated by Trypan Blue, 4′,6-diamidino-2-phenylindole (DAPI), and 3-(4,5-dimethylthiazol-2-yl)-2,5-diphenyltetrazolium bromide (MTT) assays, respectively. We have demonstrated that cancer cell viability was decreased to 55.40% when treated with FMSP-nanoparticles alone, whereas when cancer cells were treated with FMSP-nanoparticles along with crude clove extracts, the cell viability was drastically decreased to 8.50%. Both morphological and quantitative data suggest that the combination of FMSP-nanoparticles plus crude clove extracts are more effective in treating cancer cells and we suggest that the combination treatment of nanoparticles along with clove extracts hold a great promise for the cancer treatments.

## 1. Background

Breast cancer is one of the leading causes of death among women [1] and all available treatments such as chemotherapy, radiotherapy, hyperthermia, and gene therapy [2–6] do not completely control the progression of the cancer, carrying lots of side effects. Another limiting factor of current therapy is that it is based on single-mode treatment. Over the past few years, combination therapy has been successfully employed where chemotherapy is combined with radiotherapy or biotherapy combined with hyperthermia [7, 8]. In recent years, nanomedicines have been approved by the US Food and Drug Administration (FDA) for human use, and some others are undergoing clinical trials [9]. Nanoparticles have shown some exciting results in cancer cells because of their precise targeting, biocompatibility, bioavailability, and multifunctional capabilities [10–12]. Several studies have demonstrated that nanoparticles possessed anticancer properties when tested under* in vitro* and* in vivo* conditions [13–18] (Wang et al., 2009) [19–23]. Nanoparticles do carry some side effects or toxic effects when tested on high concentrations, but this problem can be solved if nanoparticles are treated along with other plant extracts. For several years, combination therapy has been among the most promising developments for obtaining high therapeutic effects with very low toxicity [24]. One of the important strategies to increase the efficacy of nanoparticles is by combining low doses of nanoparticles with either drug or plant extracts [24, 25]. Moreover, combination therapy plays a major role in minimizing drug resistance, undesired side effects, and chemoresistance, which are critical problems in cancer therapy [26]. In the present study, we have studied the effect of fluorescent magnetic submicronic polymer nanoparticles (FMSP-nanoparticles) alone and in combination with clove extracts on human breast cancer cells (MCF-7). The main reason to use clove extracts along with nanoparticles was to examine whether clove extracts enhance the nanoparticles impact on cancer cells growth and progression. There are several reports which have demonstrated that clove extracts have strong anticancer properties [27–30]. We have used different concentrations of FMSP-nanoparticles alone and in combination with clove extracts at different time intervals (24 hr and 48 hr) and evaluated their cytotoxic effects by both morphometric and quantitative methods.

## 2. Materials and Methods

### 2.1. Synthesis and Characterization of FMSP-Nanoparticles

FMSP-nanoparticles were prepared according to a previously described [31]. In brief, an organic ferrofluid which composed of iron oxide nanoparticles was stabilized in octane which was surrounded by oleic acid. First deionized water was added to the anionic magnetic emulsion and the mixture was homogenized. After that, the supernatant was detached, and the magnetic droplets were then added in deionized water. Then deionized water was added and polyethyleneimine solution was added and, after 15 mins of continuous stirring, the magnetic droplets were washed with deionized water. The amount of polyethyleneimine was adsorbed onto the magnetic droplets and was construed by using specific amine titration. The obtained fluorescent magnetic nanoparticles were then quantified by using a fluorescence spectrophotometer (LS-50 System, Perkin Elmer). Characterization of FMSP-nanoparticles was performed according to a previously described method [31]. In brief, the structure and morphology of FMSP-Nanoparticles were examined by scanning electron microscopy (SEM) (FEI, INSPECT S50, Check Republic), and the size of fluorescent submicron magnetic nanoparticle was measured by transmission electron microscopy (TEM) (FEI, MORGAGNE.68, Check Republic) respectively.

### 2.2. Extraction of Clove

Whole cloves were purchased from local markets in Dammam, Saudi Arabia, which weree manufactured by Muntazah Food Industries, Saudi Arabia. Clove was dried and ground into fine powder and fine powder of clove (4.0 grams) was dissolved in 25 mL of 70% ethanol. Dissolved mixture was then processed under sonicator (50 amplitude) for 10 minutes. The mixture was kept in the dark for 24 hours at room temperature, wrapped with aluminium foil to avoid evaporation and exposure to sunlight was avoided. The mixture was filtered through Whatman no. 1 filter paper and kept it in incubator at 37°C till ethanol had completely evaporated from mixtures. After that ethanolic clove samples were dissolved in phosphate buffer saline, pH 7.4, and processed for autoclave for 20 minutes.

### 2.3. Cell Culture and Treatments

MCF-7 is a breast cancer cell line with passage number 46 obtained from Dr. Khaldoon M. Alsamman, Clinical Laboratory Science, College of Applied Medical Science, Imam Abdulrahman Bin Faisal University, Dammam, Saudi Arabia. MCF-7 cells were cultured in T25 flask containing the DMEM media containing L-glutamine, 10% FBS, selenium chloride, 120 U/mL penicillin, and 120 *μ*g/mL streptomycin at 37°Celsius in 5% CO_2_ incubator (Heracell 150i, Thermoscientific). The cells were then seeded into 6-well, 96-well cell culture plates, and 8-chamber slides to be used for FMSP-nanoparticles and clove extract treatments. The cells with more than 80% confluence were used for the treatments. Before treatments, both FMSP-nanoparticles and clove extracts were autoclaved for 30 min to remove the contamination from the sample. The cancer cells were treated to different concentrations of FMSP-nanoparticles (1.25 *μ*g/mL, 12.5 *μ*g/mL, and 50 *μ*g/mL; 75 *μ*g/mL; 100 *μ*g/mL) alone and in combination with clove extracts (50 *μ*g/mL). The treated cells were analyzed after 24 hrs and 48 hrs intervals. For all the treatments, triplicate samples were taken for statistical calculations.

### 2.4. Cell Morphology

At the end of each treatment (24 hrs and 48 hrs), the MCF-7 cells from different treatments were removed from the incubator and were observed under an inverted microscope (TS100F Eclipse, Nikon) equipped with a digital camera. Each sample was observed under 10x, 20x, and 40x magnification and the cell morphology of control samples and treated samples were observed, and their images were recorded for the analysis.

### 2.5. Trypan Blue Staining

Trypan blue staining was performed to quantify the number of MCF-7 dead cells due to nanoparticle or nanoparticle + clove extract treatments. The cells were first washed with phosphate buffered saline (PBS) and then stained with Trypan blue (MPXX Biochemicals, Germany) prepared in a 0.4% solution in PBS. After 20 mins, the samples were processed for manual counting using a haematocytometer under an inverted microscope (TS100F Eclipse, Nikon). The difference between live cells and dead cells (blue colour) was calculated and compared in both FMSP-nanoparticles and FMSP-nanoparticles + clove extract treated groups, respectively.

### 2.6. MTT Assay

The MCF-7 cells were first cultured in the media containing DMEM supplemented with 10% FBS and 1% penicillin (100 IU/mL) and streptomycin (100 *μ*g/mL) in the CO_2_ incubator with 37°Celsius and 5% CO_2_ condition. Cells with 6×10^4^ cells/mL concentration were seeded in 96-well cell culture plates and incubated in CO_2_ incubator. Upon reaching 80% confluency, cancer cells were treated to different concentrations of FMSP-nanoparticles (1.25 *μ*g/mL, 12.5 *μ*g/mL, and 50 *μ*g/mL) alone and in combination with clove extracts (1 *μ*g/mL, 20 *μ*g/mL, 30 *μ*g/mL). In control group, no FMSP-nanoparticles or clove extracts were not added. After 48 hr treatment, 20 *μ*L of MTT (5 mg/mL) was added to each well and incubated for 4 hr. The media were changed with DMSO and each well was measured by using an ELISA plate reader (Synergy NEO2, Biotek Instruments, Winooski, VT, USA) at 570 nm wavelength. Percentage (%) of cell viability (%) was calculated as per the given formula:

(1) %  of  Cell  viability=Optical  density  of  Nanoparticles-treated  cellsOptical  density  of  control  cells×100

### 2.7. Statistical Analysis

All data were presented as mean ± standard deviation from triplicate experiments. The difference between control and FMSP-nanoparticles was evaluated by Student's* t*-test where p-values were calculated by Student's* t*-test (^*∗*^p<0.05, ^*∗∗*^p<0.01 and ^*∗∗∗*^p<0.001).

## 3. Results

### 3.1. Characterization of FMSP-Nanoparticles

**Figure 1:**
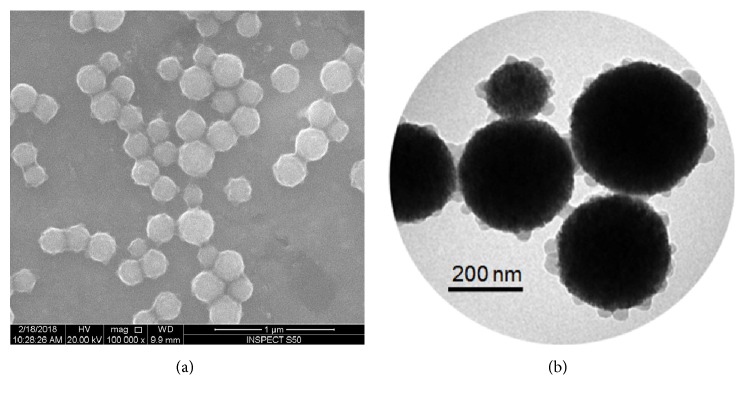
(a) Structure of fluorescent magnetic submicronic polymer FMSP-nanoparticles through Scanning Electron Microscope (SEM) with 100,000 x magnification. (b) Structure of fluorescent magnetic submicronic polymer FMSP-nanoparticles through Transmission Electron Microscope (TEM) with 70,000 x magnification.

The structure of FMSP-nanoparticles was determined by both SEM and TEM analysis. The nanoparticles appeared to be crystallized, and spherical in shape (Figure 1(a)) and TEM analysis revealed nanoparticles have an average diameter of 100 nm to 300 nm (Figure 1(b)).

### 3.2. FMSP-Nanoparticles Inhibits Cell Viability

**Figure 2:**
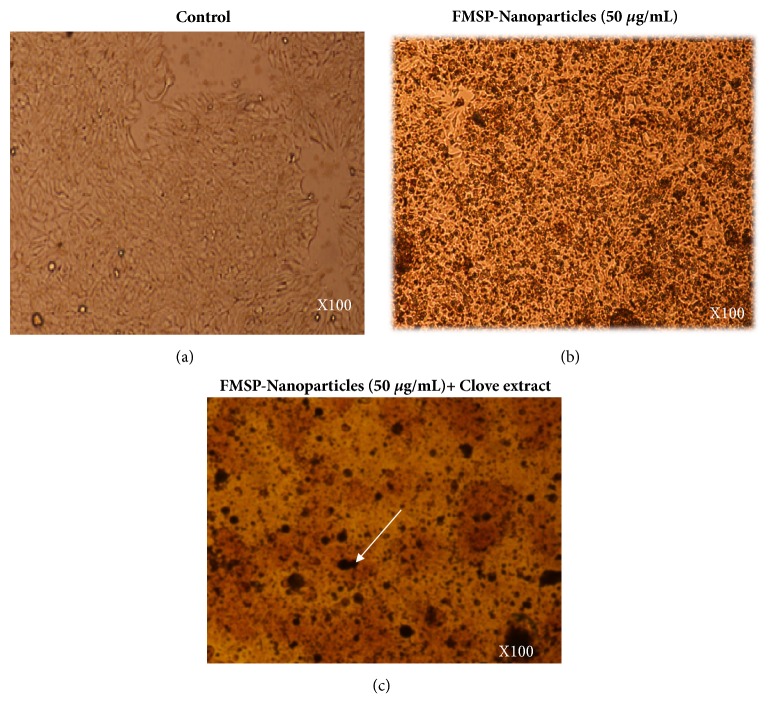
Cell morphology after treatment of FMSP-Nanoparticles alone: the MCF-7 cells were analyzed 48 hrs posttreatment. Figure (a) is control; Figure (b) is treated with FMSP-nanoparticles (50 *μ*g/mL); and Figure (c) is treated with FMSP-nanoparticles (50 *μ*g/mL) + clove extracts (100 *μ*g/mL). Arrows indicate cell dead cell debris. 100 x magnifications.

**Figure 3:**
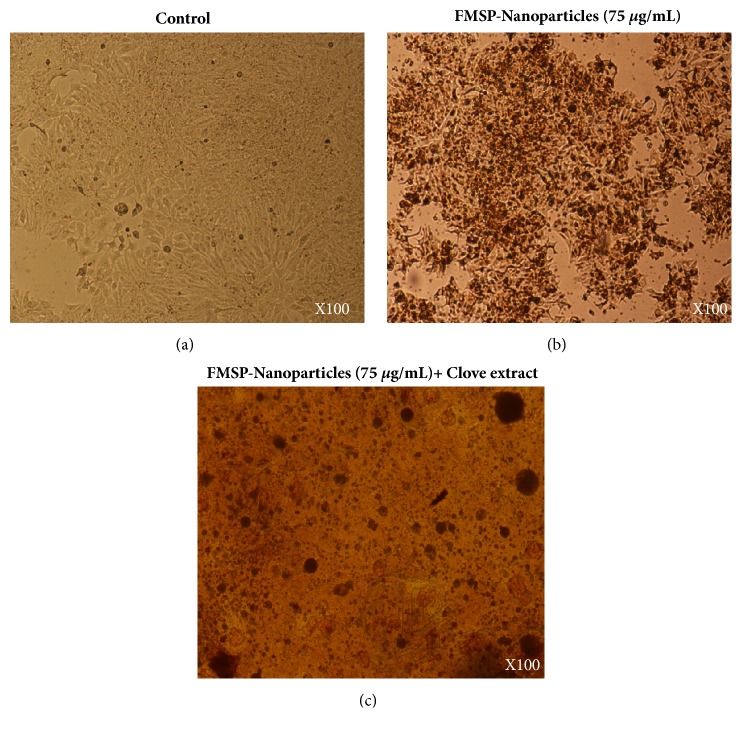
Cell morphology: the MCF-7 cells were analyzed 48 hrs posttreatment. Figure (a) is control; Figure (b) is treated with FMSP-nanoparticles (75 *μ*g/mL); Figure (c) is treated with FMSP-nanoparticles (75 *μ*g/mL) + clove extracts (100 *μ*g/mL). Arrows indicate cell dead cell debris. 100 X magnifications.

**Figure 4:**
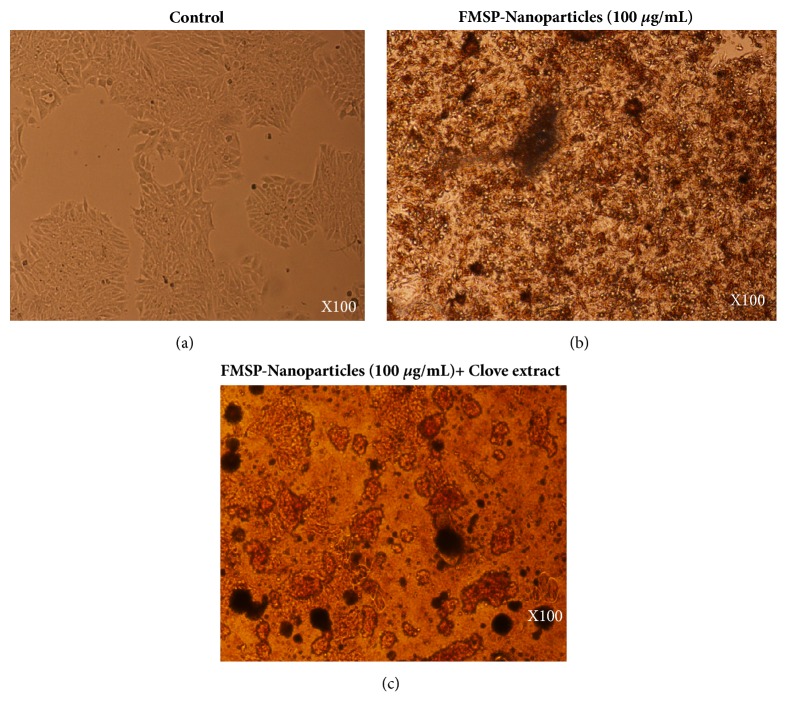
Cell morphology: the MCF-7 cells were analyzed 48 hrs posttreatment. Figure (a) is control; Figure (b) is treated with FMSP-nanoparticles (100 *μ*g/mL); Figure (c) is treated with FMSP-nanoparticles (100 *μ*g/mL) + clove extracts (100 *μ*g/mL). Arrows indicate cell dead cell debris. 100 X magnifications.

With a view to check the impact of FMSP-nanoparticles alone on cancer cells, we have evaluated the treated cells both morphologically and quantitatively. We found that FMSP-nanoparticles-treated cells showed dose-dependent response. The lower dose (1.25 *μ*g/mL) has not significantly affected the cell viability compared to control group (Figure 6), whereas the dosages of (12.5 *μ*g/mL, 50 *μ*g/mL, 75 *μ*g/mL, and 100 *μ*g/mL) caused dose-dependent decreased in the cell viability (86.14%, 77.54%, 64.00%, and 55.40%), respectively (Figure 6). We have also examined the cell morphology of FMSP-nanoparticles-treated cancer cells under microscope. Treated cells were observed in 100x, 200x, and 400x magnifications to evaluate morphological changes in the nanoparticle-treated cells. The morphology of control group cells remained normal and healthy during the testing phase (Figures 2(a), 3(a), and 4(a)). The dose of 1.25 *μ*g/mL did not show any morphological changes, whereas the dosages of 12.5 *μ*g/mL, 50 *μ*g/mL, and 75 *μ*g/mL showed strong morphological changes in the cell structure, cell membrane, and cell viability (Figures 2(b), 3(b), and 4(b)). There was an indication of nuclear disintegration, nuclear condensation, and nuclear fragmentation after the treatments. Also, few cells were found to be floated in the media (Figure 3(b)). We also observed many dead cells and their debris in the culture media (Figure 4(b)).

### 3.3. Clove Extracts Potentiate FMSP-Nanoparticles Inhibition on Cell Viability

We have examined the combined effect of FMSP-nanoparticles in combination with clove extracts alone on cancer cells using both morphometric and quantitative analyses. Like FMSP-nanoparticles alone treated cells, FMSP-nanoparticles+clove extracts also showed dose-dependent response. The lower dose of nanoparticles** (**1.25 *μ*g/mL)+clove extracts (1.25 *μ*g/mL) caused decreases in cell viability to 75.70% with compared to control group (Figure 6), whereas the dosages of (12.5 *μ*g/mL, 50 *μ*g/mL, 75 *μ*g/mL, 100 *μ*g/mL) caused dose-dependent decreased in the cell viability (55.35%, 30.85%, 20.40%, and 8.50%), respectively (Figure 6). With a view to understand the impact of FMSP-nanoparticles along with clove extracts on cancer cell structure and morphology, we have examined the cell morphology under microscope using 100x, 200x, and 400x magnifications. The morphology of control group cells remained normal (Figures 2(a), 3(a), and 4(a)) and healthy during the testing phase. The dose of nanoparticles 1.25 *μ*g/mL and 12.5 *μ*g/mL with 100 *μ*g/mL of clove extracts showed minor morphological changes in cell structure, whereas the dosages of 50 *μ*g/mL, 75 *μ*g/mL, and 100 *μ*g/mL along with 100 *μ*g/mL of clove extracts showed strong morphological changes in the cell structure, cell membrane, and cell viability (Figures 2(c), 3(c), and 4(c)). Most striking observations were complete losses of cells and their organelles (Figure 4(c)). We also observed many dead cells and their debris in the culture media (Figure 4(c)). Percentage of cell viability of FMSP-nanoparticles + clove extracts treated cells was significantly lower than FMSP-nanoparticles alone treated cells (Figure 6).

### 3.4. FMSP-Nanoparticles Induced Apoptosis in MCF-7 Cells

**Figure 5:**
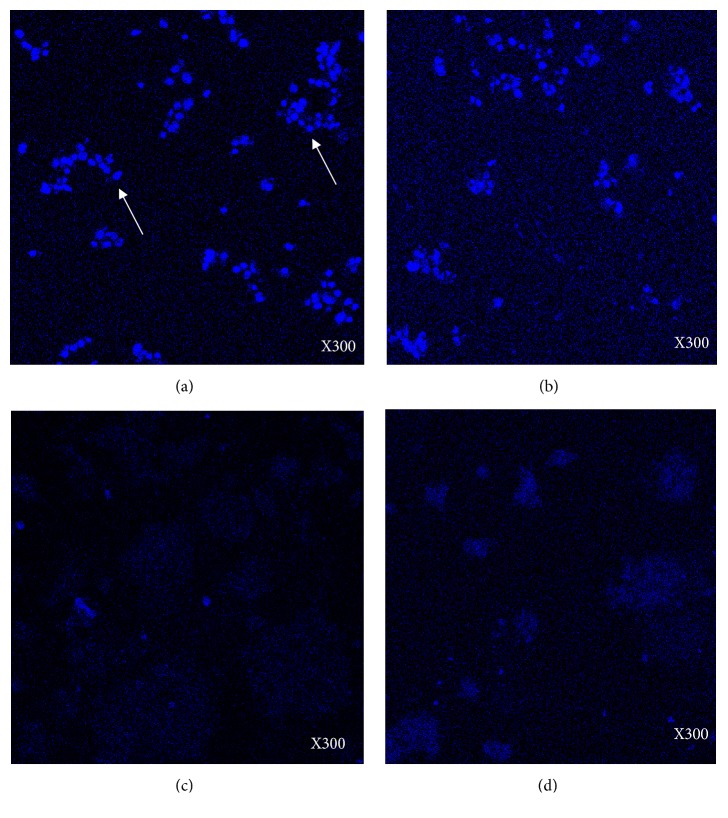
Scanning Microscopy through DAPI stained cells: the MCF-7 cells were stained with DAPI after 48 hrs of treatments. Figure (a) is control, live cells stained with blue (arrows); (b) treated with FMSP-nanoparticles (12.5 *μ*g/mL) +clove extracts (100 *μ*g/mL); (c) treated with FMSP-nanoparticles (50 *μ*g/mL) +clove extracts (100 *μ*g/mL); (d) treated with FMSP-nanoparticles (100 *μ*g/mL) +clove extracts (100 *μ*g/mL); 300 x magnifications.

**Figure 6:**
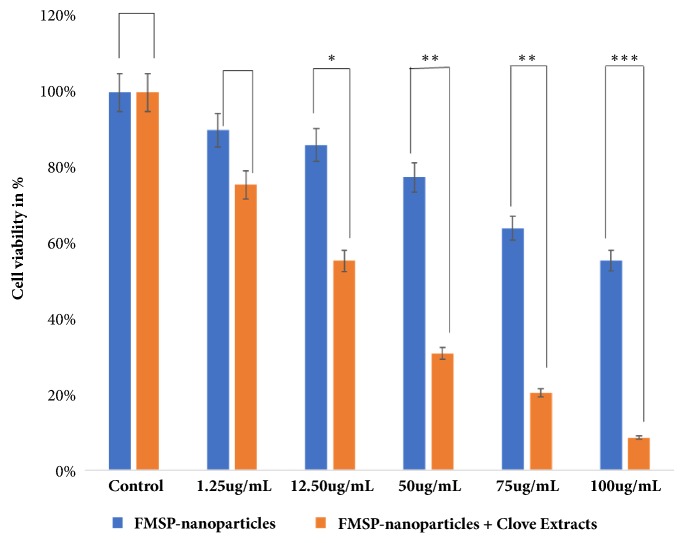
Cell viability analysis by MTT Assay. The MCF-7 cells were treated with FMSP-nanoparticles alone and with clove extracts. FMSP-nanoparticles with concentrations 1.25 *μ*g/mL, 12.50 *μ*g/mL, 50 *μ*g/mL, 75 *μ*g/mL, and 100 *μ*g/mL, whereas clove extracts with concentration 100 *μ*g/mL were used for 48 hrs. Data are the means ± SD of three different experiments. Differences between two treatment groups were analyzed by Student's* t*-test where ^*∗*^p<0.05, ^*∗∗*^p<0.01; ^*∗∗∗*^p<0.001; p-values were calculated by Student's* t*-test.

With a view to understand the cause of cell death, cancer cells treated with the combined effect of FMSP-nanoparticles and clove extracts were stained with DAPI 48 hrs posttreatment. We have found that control group cells showed the normal cell profile and DAPI-positive cells (Figure 5(a)), whereas doses of (12.5 *μ*g/mL) FMSP-nanoparticles and (100 *μ*g/mL) clove extracts treated cells showed a little less of DAPI stained cells (Figure 5(b)). The dosages of 50 *μ*g/mL and 100 *μ*g/mL FMSP-nanoparticles and (100 *μ*g/mL) clove extract treated cells showed significant loss of staining after 48 hrs (Figures 5(c) and 5(d)).

## 4. Discussion

We have reported that the combination of FMSP-nanoparticles along with clove extracts has a profound effect in reducing the cancer cell survivability and cell proliferation. Both FMSP-nanoparticles and cloves (Syzyium aromaticum) have a wide range of pharmacological and biochemical effects, including anti-inflammatory, antihyperlipidemic, and hypoglycaemic effects [32, 33]. Interestingly, there is disparity in the effect of clove extracts on cancer cells; in one study, it was reported that an extract of clove induces apoptosis in Panc-28 cells [34], whereas, in another study, it was demonstrated that an extract of clove was unable to increase ROS production in HT-29 cells [35]. But in the current study, we have shown that treatment of clove extract in combination with FMSP-nanoparticles enhanced the cancer cell death compared to cell death when FMSP-nanoparticles and clove extracts treated separately.

The objective of our study to investigate whether an extract of clove potentiates the response of FMSP-nanoparticles on the cancer cells. Our study provides further evidence that a combination of extract of clove with FMSP-nanoparticles is more effective than FMSP-nanoparticles alone treated cells. There are several studies which have been reported that combining clove extracts with 5-fluorouracil-caused a synergistic effect on cancer growth inhibition [36] (Wei et al., 2012). While there is no report on the use of clove extracts to potentiate any nanoparticles responses on cancer cells, this is first report where we have demonstrated that clove extracts enhanced FMSP-nanoparticles mediated cell death in cancer cells.

Inhibition of cell growth progression in cancer cells is one of the most effective approaches for the control of tumour growth [37]. Our finding showed that the significant decrease in the cell proliferation after 24 hrs and further decrease after 48 hrs posttreatment in a dose-dependent manner. It would be interesting to study the cell cycle progression after combined treatment of clove extracts with FMSP-nanoparticles. Moreover, it has been previously shown that treatment of an extract of clove caused a significant increase of G0/G1 phase cells was accompanied by a decrease of cells in the S and G2/M phases, respectively [29]. While we do not know the molecular mechanism behind such strong combined effects on cancer cells, the synergistic effect of clove extracts on FMSP-nanoparticles cannot be ruled out. It would be interesting to study the cancer cell death by blocking the response of the combined effects of clove extracts and FMSP-nanoparticles on cancer cells by using specific antagonists.

The size and concentration of nanoparticles play very crucial role in inducing therapeutic effects on cancer cells and variations in the concentration and sizes of nanoparticles do produce inconsistent effects. Most of the studies have used different concentrations of nanoparticles ranging from 10 ug/ml to 200 ug/ml to obtain desirable therapeutic effects [38–43]. The size of the nanoparticles is used ranged between 10 nM and 200 nM on the cancer cells [38, 42]. The nanoparticles with size 100 nm and above do not give any desirable therapeutic effects with low concentration, whereas high concentration gives good therapeutic response but poses serious health and safety problems. In the present study, we have shown that low concentration of FMSP-nanoparticles in combination with clove extracts produced a profound impact on the cancerous cells.

Besides quantitative analysis, we were also interested to know how FMSP-nanoparticles and clove extracts induce morphological changes on cancer cells. During morphometric analysis, we found that FMSP-nanoparticles treated with clove extract caused significant nuclear fragmentation and disintegration. The nuclear fragmentation and disintegration are an indication of programmed cell death, as we have seen during quantitative analysis that there was a significant cell death after combined treatment of FMSP-nanoparticles and clove extract. Both morphometric and quantitative analyses confirmed that FMSP-nanoparticles in combination with clove extracts induced concentration-dependent effects on the cancer cells. There are several reports which demonstrated that nanoparticles such as silver nanoparticles showed a strong inhibitory effect on the growth of lung tumour cells, human tongue squamous carcinoma, human breast cancer cells, and chronic myeloid leukaemia cells (He et al., 2017) [44, 45]. Both Trypan blue and MTT assays results confirm that FMSP-nanoparticles induced a dose-dependent cytotoxic effects on MCF-7 cells. These results confirm that FMSP-nanoparticles have dose-dependent effects on the cancer cells death. We do not know the mechanism of action of FMSP-nanoparticles in eliciting cancer cell death; the possibility of apoptotic pathways cannot be ruled.

## 5. Conclusion

In the present study, we have demonstrated that FMSP-nanoparticles in combination with clove extracts induced concentration-dependent cell death of cancer cells. When FMSP-nanoparticles treated alone, the dose of 1.25 *μ*g/mL decreases cell survivability to 90.03%, whereas those of 12.5 *μ*g/mL, 50 *μ*g/mL, 75 *μ*g/mL, and 100 *μ*g/mL caused decrease of cell survivability to 86.14%, 77.54%, 64.00%, and 55.40%, respectively. Interestingly, when FMSP-nanoparticles were treated in combination with clove extracts, the dose of (1.25 *μ*g/mL +100 *μ*g/mL) decreased cell survivability to 75.00%, whereas a dose of 12.5 *μ*g/mL+100 *μ*g/mL, 50 *μ*g/mL+100 *μ*g/mL, 75 *μ*g/mL+100 *μ*g/mL, and 100 *μ*g/mL+100 *μ*g/mL caused decrease of cell survivability to 55.35%, 30.85%, 20.40%, and 8.50%, respectively. In the study, we have demonstrated that the combination of FMSP-nanoparticles along with clove extracts profoundly decreases the cell survivability of cancer cells in a dose-dependent manner. Finally, we suggest that the use of combination approach is the most effective way to treat cancer cells and FMSP-nanoparticles along with clove extracts hold strong hope for the patients who are suffering from cancer.

## Data Availability

The data used to support the findings of this study are available from the corresponding author upon request.

## Abbreviations

ROS: Reactive oxygen species
HT-29: Human colorectal adenocarcinoma cell line
MCF-7: MCF-7 is a breast cancer cell line. MCF-7 is the acronym of Michigan Cancer Foundation-7
CO_2_: Carbon dioxide
DAPI: 4′,6-Diamidino-2-phenylindole
DMSO: Dimethyl sulfoxide
DMEM: Dulbecco's Modified Eagle Medium
ELISA: Enzyme-linked immunosorbent assay
FBS: Fetal bovine serum
IU: International Unit
FMSP-nanoparticles: Fluorescent magnetic submicronic polymer nanoparticles
MNPs: Magnetic nanoparticles
MTT: 3-(4,5-dimethylthiazol-2-yl)-2,5-diphenyltetrazolium bromide
nm: Nanometre
NPs: Nanoparticles
OD: Optical density
SEM: Scanning electron microscopy
TEM: Transmission electron microscopy
*μ*g: Microgram
*μ*l: Microliter.
